# Circulating microRNAs as blood-based markers for patients with primary and metastatic breast cancer

**DOI:** 10.1186/bcr2766

**Published:** 2010-11-03

**Authors:** Carina Roth, Brigitte Rack, Volkmar Müller, Wolfgang Janni, Klaus Pantel, Heidi Schwarzenbach

**Affiliations:** 1 Institute of Tumor Biology, University Medical Center Hamburg-Eppendorf, Martinistraße 52, 20246 Hamburg, Germany; 2 First Department of Obstetrics and Gynecology, Ludwig Maximilians University of Munich, Munich, Germany; 3 Clinic of Gynecology, University Medical Center Hamburg-Eppendorf, Hamburg, Germany; 4 Clinic of Gynecology, Heinrich Heine University, Düsseldorf, Germany

## Abstract

**Introduction:**

MicroRNAs (miRs) are interesting new diagnostic targets that may provide important insights into the molecular pathogenesis of breast cancer. Here we evaluated, for the first time, the feasibility and clinical utility of circulating miRs as biomarkers for the detection and staging of breast cancer.

**Methods:**

The relative concentrations of breast cancer-associated miR10b, miR34a, miR141 and miR155 were measured in the blood serum of 89 patients with primary breast cancer (M0, *n *= 59) and metastatic disease (M1, *n *= 30), and 29 healthy women by a TaqMan MicroRNA Assay.

**Results:**

The relative concentrations of total RNA (*P *= 0.0001) and miR155 (*P *= 0.0001) in serum significantly discriminated M0-patients from healthy women, whereas miR10b (*P *= 0.005), miR34a (*P *= 0.001) and miR155 (*P *= 0.008) discriminated M1-patients from healthy controls. In breast cancer patients, the changes in the levels of total RNA (*P *= 0.0001), miR10b (*P *= 0.01), miR34a (*P *= 0.003) and miR155 (*P *= 0.002) correlated with the presence of overt metastases. Within the M0-cohort, patients at advanced tumor stages (pT3 to 4) had significantly more total RNA (*P *= 0.0001) and miR34a (*P *= 0.01) in their blood than patients at early tumor stages (pT1 to 2).

**Conclusions:**

This pilot study provides first evidence that tumor-associated circulating miRs are elevated in the blood of breast cancer patients and associated with tumor progression.

## Introduction

The development of breast cancer is a complex multistep process associated with numerous genetic alterations, downregulation of tumor suppressor genes, upregulation of oncogenes and early hematogeneous dissemination of tumor cells [1-3]. Accordingly, the elucidation of the molecular mechanisms in breast cancer has been the subject of extensive research over the past decade. The search for sensitive, non-invasive markers that represent tumor-associated changes in the peripheral blood might facilitate early detection of breast cancer as well as monitoring of tumor progression and treatment responses.

MicroRNAs (miRs) are small, non-coding RNA molecules and consist of approximately 22 nucleotides. MiRs modulate post-transcriptionally the expression of numerous genes, such as of tumor suppressor genes, by binding sequence specifically to their target mRNA and inhibiting their translation into polypeptides. Moreover, miRs are involved in the regulation of different cellular processes, for example, apoptosis, hematopoietic cell differentiation, metabolism, neural development and metastasis [4,5]. The importance of miRs in the regulation of these processes has been documented by the results obtained in knockout mice. Knocking out the enzyme Dicer, responsible for the maturation of miRs, caused death of a mouse embryo [6]. As half of human miRs are located in fragile chromosomal regions harboring DNA amplifications, deletions or translocations, their expression is frequently deregulated during tumor development, which contributes to tumor progression [7].

Apart from their release of DNA and RNA, apoptotic and necrotic cells of the primary tumor also discharge miRs into the blood circulation. Protected from the degradation by endogenous RNase activity, miRs circulate in a remarkably stable form in human blood [8]. In the year 2008, extracellular serum miRs were for the first time described for patients with diffuse B cell lymphoma [9]. Although numerous publications have reported on the elevated levels of circulating nucleic acids in the blood of breast cancer patients [10-13], there are only a few publications dealing with circulating miRs in peripheral blood of breast cancer patients [7,14-16]. To date, most studies describe the profile of miR expression in breast cancer cell lines and primary tumor tissue [7].

In primary and metastatic breast carcinomas, high transcript levels of miR10b were detected and associated with tumor progression. Overexpression of miR10b in a non-metastatic breast cancer cell line induced invasion and metastasis [17]. Knockdown of miR34a by small interfering RNA significantly suppressed proliferation in the breast cancer cell line MCF-7, indicating that miR34a overexpression may be an acquired feature during carcinogenesis and support cell proliferation in breast tumors [18]. Consistent with its role in regulation of epithelial to mesenchymal transition (EMT), an essential early step in metastasis, the expression of the miR200 family, including miR200a, miR200b, miR200c, miR141 and miR429, was found to be lost in invasive breast cancer cell lines with a mesenchymal phenotype. Expression of these miRs was also lost in areas of metaplastic breast cancer specimens lacking the adhesion molecule E-cadherin [19]. Knockout mice, which do not express miR155, showed that this miR played an important role in the immune system. In Hodgkin's lymphoma, diffuse B cell lymphoma and breast cancer miR155 was highly expressed [20,21].

In summary, miRs are involved in the molecular pathogenesis of malignant tumors including breast cancer, and they might represent novel diagnostic markers. To assess the potential of serum miRs as biomarkers in breast cancer, we here examined the transcript levels of miR10b, miR34a, miR141 and miR155 in blood serum of breast cancer patients.

## Material and methods

### Patient design and healthy controls

Patients have been invited to take part in a multicenter study (SUCCESS) and attending local hospitals in the network of Ludwig Maximilians University of Munich. During September 2005 to July 2006, blood serum was taken from 59 patients with primary breast cancer about 29 days after surgery before initiation of adjuvant therapy. The median follow-up time of this patient subgroup was three years (range 1.6 to 4.2 years). During January to April 2009, blood serum from patients with metastatic breast cancer (*n *= 30) was collected 1 to 13 years after surgery of the primary tumor. All patients analyzed had histologically proven epithelial cancer and no known comorbidities. Metastatic spread in M0 patients was excluded by chest radiology, liver ultrasound scan and bone scan. Additionally, 29 healthy women with no history of cancer and in good health based on self-report were recruited as controls. All patients and healthy controls gave their informed consent. The examination of all samples was approved by the local ethics review boards. Table 1 summarizes the clinical and histopathological factors of the breast cancer patient cohort.

**Table 1:** Patients' characteristics at the time of primary diagnosis of breast cancer and correlations of the serum RNA and miR values with these parameters

Parameters	Patients (%)	Total RNA	miR10b	miR34a	miR141	miR155
**Total**	89	Median ± Standard Deviation				
		
**Age**	56 years					
(range 31 - 82 years)						
**Distant metastasis**						
**^£^M0**	59	**^a ^5.4 ± 3.5**	**^b ^0.1 ± 0.2**	**^c ^0.4 ± 0.6**	0.00 ± 16.1	**^d ^2.4 ± 1.6**
**^$^M1**	30	**^a ^2.6 ± 1.5**	**^b ^0.4 ± 0.6**	**^c ^1.8 ± 2.7**	0.0 ± 0.3	**^d ^1.5 ± 0.9**
**^#^Tumor stage**						
**pT1-2**	27 (45.8)	**^e ^3.6 ± 3.4**	0.2 ± 0.2	**^f ^0.2 ± 0.6**	0.0 ± 0.0	2.2 ± 1.9
**pT3-4**	32 (54.2)	**^e ^7.3 ± 2.9**	0.1 ± 0.1	**^f ^0.6 ± 0.5**	0.0 ± 0.0	3.1 ± 1.3
**^#^Lymph node metastasis**						
**N0**	21 (35.6)	7.3 ± 5.0	0.3 ± 0.2	0.6 ± 0.4	0.0 ± 0.01	2.2 ± 1
**N1-3**	38 (64.4)	5.3 ± 2.1	0.1 ± 0.2	0.3 ± 0.7	0.0 ± 0.01	2.6 ± 1.9
**^#^Grading**						
**II**	26 (44.1)	3.4 ± 2.7	0.1 ± 0.3	0.3 ± 0.2	0.0 ± 0.02	1.6 ± 1
**III**	33 (55.9)	5.5 ± 3.8	0.1 ± 0.2	0.5 ± 0.7	0.0 ± 0.05	2.9 ± 1.7
**^#^Estrogen receptor status**						
**positive**	37 (62.7)	5.0 ± 3.3	0.1 ± 0.3	0.3 ± 0.3	0.0 ± 0.01	2.1 ± 1.2
**negative**	22 (37.3)	5.4 ± 4.4	0.2 ± 0.2	0.4 ± 0.7	0.0 ± 0.1	2.9 ± 1.8
**^#^Progesterone receptor status**						
**positive**	33 (55.9)	5.0 ± 3.1	0.1 ± 0.3	0.3 ± 0.2	0.0 ± 0.05	2.1 ± 1.1
**negative**	26 (44.1)	5.4 ± 3.9	0.1 ± 0.2	0.4 ± 0.7	0.0 ± 0.02	2.9 ± 1.8
**^#^HER2**						
**positive**	30 (53.6)	3.9 ± 2.3	0.2 ± 0.3	0.3 ± 0.6	0.0 ± 0.01	2.4 ± 1.9
**negative**	26 (46.4)	6.0 ± 4.4	0.1 ± 0.2	0.6 ± 0.5	0.0 ± 0.1	2.9 ± 1.3
**Number of CTC^¥^**						
**positive**	47 (82.5)	2.9 ± 5.6	0.3 ± 0.3	0.5 ± 0.6	0.0 ± 0.0	2.2 ± 0.9
**negative**	10 (17.5)	5.5 ± 2.9	0.1 ± 0.2	0.3 ± 0.4	0.0 ± 0.0	2.5 ± 1.7

^a^p = 0.0001, ^b^p = 0.014, ^c^p = 0.003, ^d^p = 0.002, ^e^p = 0.0001, ^f^p = 0.01

^£^M0, patients with localized breast cancer

**^$^**M1, patients with metastatic breast cancer

^#^of M0 patients

^¥^CTC, circulating tumor cells

p values as determined by Mann and Whitney-U test.

### Cell culture

For investigations of miR expression the breast cancer cell lines MDA-MB-231 and GI-101 and the micrometastatic breast cancer cell lines BC-M1 and BC-S1 were used. MDA-MB-231 and GI-101 were cultured in DMEM (Invitrogen, Karlsruhe, Germany) supplemented with 10% FCS (fetal calf serum; PAA Laboratories, Cölbe, Germany), 2 mMol L-glutamin (Invitrogen) and 200 U/mL antibiotic-antimycotic solution (PAA). Micrometastatic cells were cultured in RPMI (Invitrogen) with 2 mMol L-glutamin (Invitrogen), 200 U/mL antibiotic-antimycotic solution (PAA), 1× Insulin-Transferrin-Selenium-A (Gibco, Eggenstein, Germany), 50 ng/mL human EGF (epidermal growth factor; Macs Miltenyi Biotec, Bergisch Gladbach, Germany) and 10 ng/mL human bFGF (basic fibroblast growth factor; Macs Miltenyi Biotec).

### Extraction of total RNA

For isolation of total RNA from human blood serum and cultured cell lines, the *mir*Vana PARIS kit (Ambion, Darmstadt, Germany) was used. Four hundred μL of serum samples and 1 × 10^6 ^lysed cells were incubated with an equal volume of Denaturation Solution for five minutes on ice. According to the manufacturer´s protocol, the RNA extraction was performed by acid-phenol:chloroform, and the precipitation was carried out by ethanol and a filter cartridge. The extracted RNA was eluted in 100 μL of preheated Elution Solution and measured on a NanoDrop ND-1000 Spectrophotometer (Thermo Scientific, Wilmington, DE, USA). The RNA samples were immediately stored at -80°C and within few days converted into cDNA.

### Conversion of total RNA into cDNA

Reverse transcription was performed by the TaqMan MicroRNA Reverse Transcription Kit (Applied Biosystems, Darmstadt, Germany). The 10 μL-reverse transcription reaction contained 0.1 μL 100 mM dNTPs, 0.66 μL MultiScribe Reverse Transcriptase (50 U/μL), 1 μL 10× Reverse Transcription Buffer, 0.13 μL RNase Inhibitor (20 U/μL), nuclease-free water and 3.33 μL or 1 μL RNA derived from human serum or cultured cells, respectively. On a MJ Research PTC-200 Peltier Thermal Cycler (Global Medical Instrumentation, Ramsey, Minnesota, USA) the reaction was carried out at 16°C for 30 minutes, 42°C for 30 minutes and 85°C for 5 minutes.

### Preamplification of miR141 and miR16 cDNA

Due to the low expression of miR141, a preamplification of its cDNA was performed. To effectively normalize the expression data of miR141, cDNA of the reference miR16 was also preamplified. Both cDNAs were preamplified in 7.5 μL Taq PCR Mastermix and 0.75 μL 20 × TaqMan MiRNA Assay mix by using the Taq PCR Mastermix Kit (Qiagen, Hilden, Germany). The PCR was run on a MJ Research PTC-200 Peltier Thermal Cycler (Global Medical Instrumentation): 1 cycle at 95°C for 5 minutes, 15 cycles at 95°C for 20 s, 60°C for 20 s and 72°C for 20 s, and a terminal cycle at 72°C for 5 minutes.

### Quantitative real-time PCR of miR10b, miR34a, miR141 and miR155

For quantitative real-time PCR, the miR-specific TaqMan MicroRNA Assays (Applied Biosystems) for miR16 (reference miR), miR10b, miR34a, miR141 and miR155 were used. In a 10 μL-reaction, 1 μL cDNA or 3 μL preamplified cDNA were mixed with 5 μL TaqMan Universal PCR Master Mix No AmpErase UNG and 0.5 μL miR-specific TaqMan MicroRNA Assay Mix on a twin-tec real-time PCR plate (Eppendorf, Hamburg, Germany). The quantitative real-time PCR reaction was performed at 95°C for 10 minutes and in 40 cycles at 95°C for 15 s and 60°C for 60 s on a Mastercycler Realplex (Eppendorf). Melting curve analyses were performed to verify the specificity and identity of PCR products.

The obtained data of the miR expression levels were calculated and evaluated by the ΔCt method as follows: ΔCt = mean value Ct (reference miR16) - mean value Ct (miR of interest). The relative expression of miR of interest corresponded to the 2^(ΔCt) value.

As recommended by the manufacturer (Applied Biosystems), miR16 has been chosen as reference for normalization of the expression levels of our miR panel. To effectively normalize the expression data of miR10b, miR34a, miR141 and miR155, we analyzed the miR16 expression levels and found that the levels remained relatively constant across the serum samples. We calculated a mean value of 21.12, 22.23, and 21.17 with a standard deviation of 2.10, 2.12 and 0.98 for the subgroups of M0 patients, M1 patients and healthy women, respectively.

### Statistical analysis

The statistical analyses were performed using the SPSS software package, version 18.0 (SPSS Inc. Chicago, IL, USA). The chi square or two-tailed Fischer´s exact test was used to identify potential associations of miR concentrations in blood serum with the clinical and histopathological risk factors of the breast cancer patients. For nonparametric comparisons, univariate analyses of the Mann Whitney-U test of two independent variables and bivariate analyses of the Spearman-Rho test were used. Kaplan-Meier plots were drawn on to estimate overall survival and recurrence, and the Log rank test was applied for statistical analyses. Missing data were handled by pairwise deletion. A *P*-value ≤0.05 was considered as statistically significant. All *P*-values are two-sided.

## Results

### Expression pattern of miR10b, miR34a, miR141 and miR155 in breast cancer cell lines

Before examining the signature of the four miRs (miR10b, miR34a, miR141 and miR155) in human blood, we analyzed their expression patterns in the widely used breast cancer cell lines MDA-MB-231 and GI-101 as well as in the micrometastatic cell lines BC-M1 and BC-S1 which were established from disseminated tumor cells present in the bone marrow of breast cancer patients without overt distant metastases [22]. Interestingly, BC-M1 and BC-S1 cells co-express cytokeratin and vimentin consistent with an EMT-like invasive phenotype as well as other cancer stem cell characteristics [23,24].

**Figure 1:**
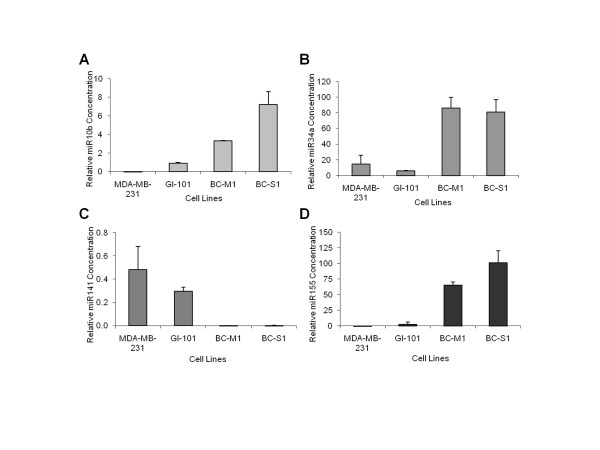
**MiR expression in breast cancer cell lines as determined by quantitative real-time PCR**. Basal expression levels of miR10b **(A)**, miR34a **(B)**, miR141 **(C) **and miR155 **(D) **in breast cancer cell lines MDA-MB-231 and GI-101, and micrometastatic breast cancer cell lines BC-M1 and BC-S1. The relative transcript levels of the miRs were determined by the low cycle threshold (Ct) values.

We quantified the relative expression of these miRs by determining the low cycle threshold (Ct) values. As shown in Figure 1A-D, the relative values of the miRs in MDA-MB-231 and GI-101 differed from those in the micrometastatic cell lines. Compared with the low values in MDA-MB-231 and GI-101 cells, the transcript levels of miR10b (Figure 1A), miR34a (Figure 1B) and miR155 (Figure 1D) were significantly upregulated in the micrometastatic breast cancer cells BC-M1 and BC-S1. MiR141 expression was clearly upregulated in MBD-MB-231, and GI-101 cells, whereas it was nearly abolished in the micrometastatic cells (Figure 1C).

### Profiling of total RNA, miR10b, miR34a, miR141 and miR155 in blood serum of breast cancer patients and healthy women

**Figure 2:**
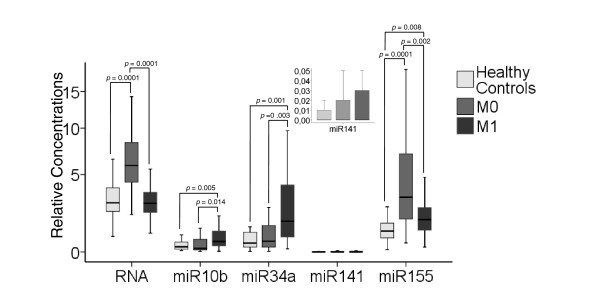
**Levels of total RNA and miRs in blood of breast cancer patients and healthy controls**. The box plot and the additionally integrated box plot of miR141 show the different, relative amounts of total RNA, miR10b, miR34a, miR141 and miR155 which circulate in blood of healthy individuals (*n *= 29), M0 patients (*n *= 59) and M1 patients (*n *= 30). The relative transcript levels of miRs were determined by the low cycle threshold (Ct) values. As determined by Mann and Whitney-U test, the significant *P-*values of the statistical evaluations of serum RNA and miR levels are indicated above the blots.

In the box plot of Figure 2, the relative quantification of circulating total RNA, miR10b, miR34a, miR141 and miR155 in blood serum of 29 healthy individuals and 89 patients with breast cancer (M0, *n *= 59 and M1, *n *= 30) are depicted. To determine the differences in the relative expression profiles, we performed univariate analyses of the Mann Whitney-U test.

As shown in Figure 2, M0 patients had significantly higher levels of circulating total RNA in their blood (normalized Ct values), which was taken approximately four weeks after surgery, than healthy individuals (*P *= 0.0001). Counter to expectation, M1 breast cancer patients had similar RNA concentrations to healthy individuals. M0 patients had on average 2.0-fold higher median values of RNA than M1 patients and healthy controls (*P *= 0.0001). In addition, we also analyzed paired serum samples taken pre- and post-operatively from 10 breast cancer patients. Our data showed similar RNA levels before and four weeks after surgery (*P *= 0.375, data not shown).

In respect to the miRs, the median values of miR155 of both subgroups M0 and M1 were 2.7-fold (*P *= 0.0001) and 1.6-fold (*P *= 0.008) increased in comparison to healthy women. In contrast, the values of miR10b (4-fold, *P *= 0.005) and miR34a (4.5-fold, *P *= 0.001) could only discriminate M1 patients, but not M0 patients, from healthy individuals. In M0 and M1 patients, their relative yields of miR141 did not differ significantly between healthy women and women with breast cancer (Figure 2).

### Correlation of circulating total RNA and miRs in serum of breast cancer patients with clinical and histopathological factors

We compared the relative concentrations of circulating total RNA, miR10b, miR34a, miR141 and miR155 in blood serum of the 89 breast cancer patients with their clinical and histopathological data. For these statistical evaluations, the Mann and Whitney-U test of the non-parametric comparison of two independent variables and Log rank test were used (Table 1). As shown in Table 1 and Figure 2, the transcript levels of miR10b (*P *= 0.014) and miR34a (*P *= 0.003) were significantly higher in serum of M1 patients than M0 patients, whereas the levels of miR155 (*P *= 0.002) and total RNA (0.0001) in M1 patients were lower than in M0 patients. Moreover, high values of total serum RNA (*P *= 0.0001) and miR34a (*P *= 0.01) correlated with advanced tumor stages within the subgroup of 59 M0 patients (Table 1).

In addition, we correlated the values of total RNA and miRs with the number of mononuclear blood cells of M0 patients, because leukocytes could also be a source for the release of these RNAs. Unfortunately, no data on the number of leukocytes were available for M1 patients. Surprisingly, M0 patients at early tumor stages had significantly more leukocytes in their blood than patients at advanced tumors (*P *= 0.016). No significant relationship of the number of leukocytes to the RNA and miR values could be detected.

The statistical assessments between the concentrations of miRs and the other clinical and histopathological data as well as the number of circulating tumor cells (CTC) did not reach any statistical significance (Table 1). The number of CTC was only determined in blood of M0 patients. Within this subgroup, 57 of 59 patients were analyzable for CTC but only 10 (18%) patients had CTC in their blood. This low number of CTC-positive patients impeded an accurate statistical analysis.

Kaplan-Meier and Log-rank models were used to assess the prognostic potential of miR levels in serum of breast cancer patients. The median follow-up time was three years (range 1.6 to 4.2 years). Median expression levels were used for grouping the serum samples according to low and high expression. The serum levels of miR141 could not discriminate breast cancer patients from healthy women but the association with the clinical outcome reached borderline significance (*P *= 0.1 for progression-free survival in M1 patients and *P *= 0.06 for recurrence-free survival in M0 patients). The measurements of the other serum miRs were not associated with either diagnosis or progression of breast cancer.

## Discussion

Emerging evidence indicates that the deregulation of miRs might play a crucial role in breast carcinogenesis [25]. However, their precise role in promoting breast cancer progression and metastasis remains under investigation. Previous studies scrutinizing the signature of miRs in tumor patients have mainly been carried out by using tumor tissues. To date, only a handful of publications have dealt with circulating miRs in blood of breast cancer patients [7,14-16]. Based on their regulation of relevant target genes [19,21,26,27], we assembled a set of four miRs (miR10b, miR34a, miR141 and miR155) and examined whether their expression profile in blood serum was associated with diagnosis and progression of breast cancer. To our best knowledge, only miRNA10b, miR141 and miR155 have been measured in blood serum so far by other groups [9,14-16] including miRNA10b and miR155 in breast cancer [7,15,16].

In the present study, we demonstrated that cell-free serum total RNA and miR155 in M0 patients as well as miR10b, miR34a and miR155 in M1 patients may discriminate breast cancer patients from healthy individuals. Whereas M0 patients at advanced tumor stages had dramatically more RNA in their blood than patients at early tumor stages, M1 patients with overt distant metastases surprisingly displayed lower levels of RNA. This observation cannot be explained by a higher cell turnover in the advanced tumors of M0 patients because these tumors were resected four weeks before taking the blood samples for miR analysis. Notably, because we collected the serum samples postoperatively, we can not exclude that serum RNA may also stem from other tissue sources, such as mononuclear blood cells, CTC or occult micrometastases. However, the statistical evaluations of the number of leukocytes showed no association with the serum RNA concentrations in M0 patients, and an inverse correlation with increasing tumor stages. Since only 10 M0 patients had CTC in their blood, a meaningful statistical comparison between the CTC and RNA data was not possible. However, we did not even observe a tendency of higher RNA and miR levels in serum of CTC-positive M0 patients. These findings indicate that the bulk of circulating RNA may probably not stem from mononuclear blood cells, and the contribution of CTC remains questionable.

The kinetics and metabolism of circulating RNA has not yet been clearly elucidated, and a possible caveat of our study results might be that our blood serum samples were collected approximately four weeks after surgery (that is, before initiation of adjuvant therapy), which might limit their use for diagnostic purposes. To address this important concern, we analyzed paired serum samples taken pre- and postoperatively from 10 breast cancer patients. Our data showed similar RNA levels before and four weeks after surgery suggesting that the serum RNA levels did not decrease significantly after surgery of the primary breast tumor.

Considering the particular miRs in blood serum of our breast cancer patient cohort, increasing concentrations of miR10b and miR34a significantly correlated with the occurrence of overt metastasis. In respect to miR10b, early studies detected this miR to be down-regulated in breast tumor tissue compared with normal breast tissue [28,29]. However, in a recent study using plasma and serum, the expression of circulating miR10b did not differ significantly between a breast cancer cohort and healthy controls, but significantly higher levels were observed in cancer patients with ER (estrogen receptor)-negative disease than in those with ER-positive breast cancer [16]. Our analyses on serum miR10b support the studies of Ma *et al*. who reported that miR-10b specifically played a role in the metastatic process but not in primary tumor development. They found this miR to be highly expressed in metastatic breast cancer cells, and its overexpression initiated invasion and metastasis in a combination of mouse and human cell models by indirectly activating the pro-metastatic gene RhoC [17].

With regard to miR34a, discrepant data on the expression of this miR in diverse tumor entities have been described. It was reported that miR34a was downregulated in non-small cell lung carcinomas, pancreas tumor cell lines, colon carcinomas and primary neuroblastomas [27,30]. In contrast, Dutta *et al*. detected a high incidence of miR34a overexpression in various tumor types and undetectable expression in only poorly differentiated gastric adenocarcinomas and renal cell carcinomas. In the majority of carcinomas they perceived the participation of miR34a in cell proliferation [18]. Consistent with the data of Dutta *et al*. [18], we detected high miR34a levels in the blood of our breast cancer cohort, in particular in patients with advanced tumor stages and metastatic disease.

In addition, we detected significantly lower serum levels of miR155 in M1 than in M0 patients, but both subgroups had significantly higher miR155 levels than healthy individuals. Our findings are in contrast to another study showing that transcript levels of circulating miR155 in blood did not differ significantly between breast cancer and healthy control cohorts [16]. Using primary breast cancer tissues and epithelial cells, Kong *et al*. observed that miR155 may play an important role in the TGFβ-induced EMT, cell migration and cell invasion by targeting the small G-protein RhoA. They indicated that it might be a potential therapeutic target for breast cancer intervention [21]. Moreover, circulating miR155 was shown to be differentially expressed in the sera of women with hormone-sensitive and -insensitive breast cancer. Women with progesterone receptor-positive tumors had a higher miR155 expression than patients with tumors that were progesterone receptor-negative [15]. In our breast cancer cohort, we did not observe different serum miR155 levels between hormone receptor-positive and -negative patients. This discrepancy may mainly be explained by the small number of patients analyzed in that report.

MiR141 expression was reported to be associated with cancer progression in breast cancer [31]. Our present findings showed that although the levels of miR141 did not markedly change in the blood of patients with metastatic disease in comparison to patients with primary breast cancer, the quantification of the serum miR141 concentrations might have a prognostic value in particular in node-negative patients. To sustain this potential role of miR141 as prognostic marker, additional results of future prospective studies with more patients and longer follow-up periods are needed.

Finally, we assessed the transcript levels of the four miRs in tumor and micrometastatic cell lines. In tumor cell lines, the concentrations of the four miRs differed strikingly from those in the micrometastatic tumor cell lines [24]. Comparing the miR profiles of the cell lines with those observed in the blood of breast cancer patients revealed that the profile of the micrometastatic cell lines was nearly congruent with that in the blood of M1 patients, whereas the profiles of the other tumor cell lines were not concordant with those in the patient´s blood. Thus, the micrometastatic cell lines might be good models for future functional analyses.

## Conclusion

The significant increase of miR10b, miR34a and miR155 concentrations in the peripheral blood of breast cancer patients and the observed associations with tumor progression suggest a potential clinical utility of circulating miRs as a new class of future biomarkers.

## Abbreviations

Ct: cycle threshold; CTC: circulating tumor cells; EMT: epithelial to mesenchymal transition; ER: estrogen receptor; miRs: microRNAs; M0: primary breast cancer; M1: metastatic disease

## Competing interests

The authors declare that they have no competing interests.

## Authors' contributions

CR and HS performed all experiments. HS and CR performed the statistical analysis. HS drafted the manuscript and KP revised the manuscript. BR, VM and WJ prepared the clinical material. CR summarized the clinical parameters. HS, CR and KP were involved in conception and design of the study and participated in the discussion and interpretation of the results.
